# Identification of a Prognostic Signature Composed of GPI, IL22RA1, CCT6A and SPOCK1 for Lung Adenocarcinoma Based on Bioinformatic Analysis of lncRNA-Mediated ceRNA Network and Sample Validation

**DOI:** 10.3389/fonc.2022.844691

**Published:** 2022-03-28

**Authors:** Wenjun Tang, Qiaonan Lu, Jianling Zhu, Xiaowei Zheng, Na Fang, Shaoping Ji, Feng Lu

**Affiliations:** ^1^ Joint National Laboratory for Antibody Drug Engineering, The First Affiliated Hospital, School of Medicine, Henan University, Kaifeng, China; ^2^ Department of Immunology, School of Basic Medical Sciences, Henan University, Kaifeng, China; ^3^ School of Life Sciences, Tsinghua University, Beijing, China; ^4^ Department of Clinical Laboratory, Puyang Hospital of Traditional Chinese Medicine, Puyang, China; ^5^ Department of Biochemistry and Molecular Biology, School of Basic Medical Sciences, Henan University, Kaifeng, China

**Keywords:** long non-coding RNAs, competing endogenous RNA, lung adenocarcinoma, prognostic signature, ceRNA network

## Abstract

Lung adenocarcinoma (LUAD) is one of the most common malignant tumors with high morbidity and mortality in China and worldwide. Long non-coding RNAs (lncRNAs) as the competing endogenous RNA (ceRNA) play an essential role in the occurrence and development of LUAD. However, identifying lncRNA-related biomarkers to improve the accuracy of LUAD prognosis remains to be determined. This study downloaded RNA sequence data from The Cancer Genome Atlas (TCGA) database and identified the differential RNAs by bioinformatics. A total of 214 lncRNA, 198 miRNA and 2989 mRNA were differentially identified between LUAD and adjacent nontumor samples. According to the ceRNA hypothesis, we constructed a lncRNA-miRNA-mRNA network including 95 protein-coding mRNAs, 7 lncRNAs and 15 miRNAs, and found 24 node genes in this network were significantly associated with the overall survival of LUAD patients. Subsequently, through LASSO regression and multivariate Cox regression analyses, a four-gene prognostic signature composed of GPI, IL22RA1, CCT6A and SPOCK1 was developed based on the node genes of the lncRNA-mediated ceRNA network, demonstrating high performance in predicting the survival and chemotherapeutic responses of low- and high-risk LUAD patients. Finally, independent prognostic factors were further analyzed and combined into a well-executed nomogram that showed strong potential for clinical applications. In summary, the data from the current study suggested that the four-gene signature obtained from analysis of lncRNA-mediated ceRNA could serve as a reliable biomarker for LUAD prognosis and evaluation of chemotherapeutic response.

## Introduction

Lung adenocarcinoma (LUAD) is one of the most common malignant tumors with high morbidity and mortality in China and worldwide, accounting for 40% of all lung cancers (1, 2). Although tremendous progress has been achieved in diagnosis and treatment strategies in the past 10 years, the 5-year overall survival rate of patients with LUAD is still meager, less than 20% (3). The prognosis of LUAD is closely related to many factors such as lymph node metastasis, distant metastasis and diagnostic time. At present, the insufficient understanding of the biological characteristics of LUAD limits the further improvement of therapeutic effects. Therefore, it is urgent to clarify the pathogenesis of tumors and identify novel biomarkers and treatment schemes to improve the prognosis.

Long non-coding RNAs (lncRNAs) are a type of transcripts with little or no protein-coding potential, which are more than 200 nucleotides in length (4, 5). In recent years, increasing evidence has demonstrated that lncRNAs can act as the competing endogenous RNA (ceRNA) to indirectly regulate downstream target mRNA expression by competing for shared miRNAs and subsequently participate in the development of complex disease phenotypes and various pathological processes, including cancers (6–8). For example, lncRNA SHHG6-003 could bind with miR-26a/b/TAK1, promote the proliferation of hepatocellular carcinoma (HCC) cells, and shorten the overall survival of HCC patients (9). The lncRNA HOTAIR could regulate the expression of human epithelial growth factor receptor 2 (HER2) by competing for mir-331-3p, thus playing an oncogenic role in gastric pathogenesis (10). Additionally, in 2019, Wang et al. reported that the dysregulated MMP9/ITGB1-miR-29b-3p-HCP5 competing endogenous RNA (ceRNA) network was closely linked to poor prognosis of pancreatic cancer (11). In 2018, another study observed four ceRNA based on lncRNA, which had significant prognostic value in breast cancer (12). Together, these findings demonstrate that the imbalance of the lncRNA-miRNA-mRNA network is involved in the pathogenesis of various cancers. However, the overall biological role and potential molecular mechanism of the lncRNA-mediated ceRNA network in LUAD are still unclear.

In this study, LUAD-related gene expression profiles were downloaded from the TCGA database. Differentially expressed lncRNAs, miRNAs and mRNAs were analyzed using bioinformatics methods. A LUAD-specific lncRNA-miRNA-mRNA regulatory network was constructed following the ceRNA hypothesis. Then, a 4-gene prognostic signature composed of GPI, IL22RA1, CCT6A and SPOCK1 was developed and validated using the node genes of the lncRNA-mediated ceRNA network. Finally, independent prognostic factors were further analyzed and combined into a nomogram, which was confirmed to be highly accurate in predicting the survival of patients with LUAD.

## Materials And Methods

### Clinical Lung Adenocarcinoma and Adjacent Nontumorous Lung Tissues

Forty paired LUAD and adjacent nontumorous tissues were obtained from patients undergoing surgery at the First Affiliated Hospital of Henan University and Puyang Hospital of traditional Chinese medicine, China, between 2018 and 2020. This study was approved by the Ethics Committee of Medical School of Henan University, China. All methods in this study were performed following the approved guidelines. Written informed consent was obtained from each patient before sample collection.

### Cell Culture and Stable Transfection of shRNA

All NSCLC cell lines (HCI-H1299 and A549), immortalized lung epithelial cell line BEAS-2B and HEK293T were obtained from the Cell Bank of Type Culture Collection of the Chinese Academy of Sciences (Shanghai, China). The cells were cultured in DMEM medium (Corning, USA) with 10% fetal bovine serum (Pan biotechnology, Germany) at 37°C and 5% CO_2_ (all cells were cultured under the same conditions) and were not passaged more than 25 times after thawing. Cells growing at an exponential rate were used for experiments. Cells were periodically evaluated to confirm *Mycoplasma-*negative status, and cell lines were authenticated by growth characteristics, examination of morphology and short tandem repeat analysis.

CCT6A in H1299 and A549 cells were stably knockdown by transduction with pre-made lentiviral short hairpin RNA (shRNA) (TranSheepBio, Shanghai, China). The shRNA vectors included TRCN0000062514 named as sh1CCT6A (target sequence: 5’- CGTGTCATTAGAGTATGAGAA-3’) and TRCN0000062515 named as sh2CCT6A (target sequence: 5’-CCAGAACATCTCTTCGTACTA-3’). The sequences of scramble control shRNA (shNC) were 5’- TTCTCCGAACGTGTCACGTAAT-3’. According to the manufacturer’s instruction, the recombinant lentiviral vector, envelope plasmid (pMD2.G) and packaging vector (psPAX2) were co-introduced into HEK293T cells through Lipofectamine 2000. After 48 h transduction, the culture supernatant was collected and filtered with 0.45μm filter, and then was used to infect H1299 and A549. The infected cells were screened by puromycin at 9.18 μmol/L. The efficiency of gene silencing was detected by Western blot analysis.

### Colony Formation Assay and Wound-Healing Assay

Cell proliferation and migration were assessed by colony formation and wound-healing assay. Details of the relevant contents have been described previously (13)

### Immunoblotting

Tissues or cells were lysed on ice in RIPA lysis buffer containing 500 mM NaCl, 50 mM Tris pH 8.0, 1mM EDTA, 1% NP-40 and 1×cocktail of protease inhibitors (Roche, Lewes, UK). Protein concentrations were quantified according to the manufacturer’s instructions (Pierce, Rockford, IL). Protein lysates were separated by SDS-PAGE and transferred to polyvinylidene fluoride membranes. Details of the relevant content have been described previously (13). Information of antibodies used was provided in **Supplementary Table 1**.

### Analysis of Expression Profiles of lncRNAs, miRNAs and mRNAs in LUAD and Adjacent Nontumorous Tissues

Raw sequencing data of LUAD-related RNAs expression and complete clinical data of the corresponding patients were downloaded from the TCGA database (https://portal.gdc.cancer.gov/). After homogenizing the TCGA raw data using the trimmed mean of M-values (TMM) method, the expression level of RNA was converted to a log2 value. Then, the R package of edgeR was used to screen for differentially expressed lncRNAs, miRNAs and mRNAs in LUAD and adjacent nontumorous tissues. Statistical significance was defined as *P<0.05* and absolute Log Fold Change ≥1. Heatmaps and volcano plots of differentially expressed RNAs (DERNAs) were drawn using ggplots and heatmap software packages.

### Construction of a ceRNA Network in LUAD

The miRcod database (http://www.mircode.org/) was used to predict the interaction between differentially expressed lncRNA and miRNA (14). The mRNA targeted by differentially expressed miRNA were searched from the miRTarBase (http://mirtarbase.mbc.nctu.edu.tw/), miRDB (http://www.mirdb.org/), and TargetScan (http://www.targetscan.org/) databases using the Perl program (version:5.26.1) (15–17). A lncRNA-miRNA-mRNA ceRNA network based on the “ceRNA hypothesis” was established and visualized by Cytoscape software (http://cytoscape.org/).

### Establishment and Validation of the Prognostic Signature Based on the ceRNA Network

The survival-related node genes in the ceRNA network were extracted by univariate Cox regression analysis with the threshold of Hazard Ratio ≠1 and *P<0.05*. To minimize overfitting, the R package “glmnet” was used to further extract prognostic genes for following multivariate Cox regression by a least absolute shrinkage and selection operator (LASSO) regression analysis. Then, the R package “caret” was used to randomly divide TCGA_LUAD patients into training and testing cohorts (18). A prognostic signature based on the ceRNA network was constructed in the training cohort by multivariate Cox regression (19). The prognostic gene signatures were shown as risk score=sum [gene expression×coefficient]. The risk score for each patient was calculated. We divided the LUAD patients into the high-risk and low-risk groups with the median risk scores as our cut-off. To verify the prognostic value of the above ceRNA-related genes signature, survival and ROC curve analyses were performed using the testing cohort and entire cohort as the validation set. Principal components analysis (PCA) was used to explore the distribution patterns of the different risk groups. A prognostic nomogram including risk scores and clinical features for predicting the likelihood of 1-, 3-, and 5-year OS was developed by R “rms” package. The calibration curves and C-index were used to evaluate the predictive accuracy of the nomogram. Finally, the mRNA expression levels of the prognostic signature genes were validated using Oncomine (http://www.oncomine.org/), TCGA and GSE32863 databases. The protein expression of levels was further verified by western blot analysis using 40 pairs of LUAD and adjacent nontumorous tissues.

### Gene Set Enrichment Analysis

To investigate the potential biological pathways and processes of the ceRNA network-related genes signature, we conducted the KEGG pathway analysis through Gene Set Enrichment Analysis (GESA) for the training cohort, with *FDR<0.05* as a threshold for the significant pathways.

### Analysis of 22 Immune Cell Types’ Infiltration Patterns and Correlation Between the ceRNA Network-Associated Genes Signature and Biomarkers for Immunotherapy

The CIBERSORT algorithm was utilized to estimate the fraction of 22 immune cell types in the LUAD samples from gene expression data in the training cohort. Samples with a CIBERPORT output value of *P<0.05* were considered to meet the conditions for further analysis. The difference of immune cells in the proportion between the high- and low-risk groups was analyzed by Wilcoxon rank sum test. Additionally, it is worth noting that immune checkpoints are biomarkers for selecting LUAD patients for immunotherapy. Therefore, in this study, we analyzed the correlation between the ceRNA network-associated genes signature and key immune checkpoints (PD-1, PD-L1, CTLA-4, LAG3, TIM-3, TIGIT, CD80, CD276, TNFSF4 and VTCN1) using the R package “lmma”.

### Prediction of Chemotherapeutic Response Based on the ceRNA Network-Associated Genes Signature

Chemotherapy is one of the effective methods for treating advanced patients with LUAD. The clinical response of each LUAD patient in high- and low-risk groups to chemotherapy was estimated according to the Genomics of Drug Sensitivity in Cancer (GDSC) data. Nine commonly used chemotherapy drugs, cisplatin, docetaxel, doxorubicin, erlotinib, etoposide, gemcitabine, paclitaxel, vinorelbine and cytarabine, were selected for the chemotherapeutic response prediction through the ridge regression using the “pRRophetic” R package. The half-maximal inhibitory concentration (IC50) predicted of each TCGA_LUAD patient was used to assess differential chemotherapeutic response (20).

### Statistical Analysis

SPSS21.0 (SPSS Inc., Chicago, IL, USA) and R software (version 3.6.0) were used for all statistical analyses. The difference between the two groups was assessed using the Student’s t-test. The continuous data are expressed as the mean ± standard deviation (SD). All statistical tests were two-tailed, and statistical significance was set at *P<0.05*.

## Results

### Differential Expression Analyses of lncRNAs, miRNAs, and mRNAs in LUAD

We analyzed the differential expression of lncRNA, miRNA, and mRNA in LUAD by R software package using TCGA database containing 497 LUAD and 54 paracancerous samples, with *P<0.05* and absolute Log2 Fold Change of 1 as the threshold. A total of 214 lncRNA (51 down- and 163 upregulated), 198 miRNA (87 down- and 111 upregulated) and 2989 mRNA (1344 down- and 1645 upregulated) were identified between LUAD and adjacent nontumorous samples (**Supplementary Table 2**). The differentially expressed lncRNA, miRNA and mRNA distribution were visualized by volcano plots and heatmaps (**Supplementary Figure 1**).

### Establishment of the ceRNA Network in LUAD

To explore the potential regulatory mechanism of ceRNA in LUAD, we tried to establish the ceRNA network for LUAD based on the ceRNA hypothesis (19). Using miRcode, miRTarBase, and miRDB databases, a total of interactions of 119 miRAN-mRNA pairs and 15 lncRNA-miRNA pairs were identified (**Supplementary Table 3**). Finally, a LUAD-specific lncRNA-miRNA-mRNA ceRNA network, consisting of 7 lncRNAs, 15 miRNAs, and 95 mRNAs, including 117 nodes and 134 edges, was constructed and visualized (**Supplementary Figure 2**).

### Developing and Validation of the Prognostic Signature Based on the ceRNA Network

**Figure 1:**
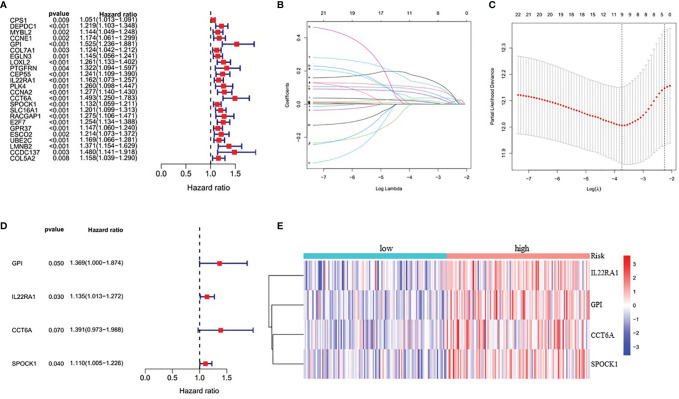
Identification of four significantly prognostic genes and their expression in LUAD. **(A)** Forest plot of node genes based on lncRNA-mediated ceRNA network by univariate Cox regression analysis. **(B)** LASSO coefficient patterns of the 24 node genes in TCGA_LUAD database. **(C)** LASSO regression with tenfold cross-validation obtained 9 prognostic genes using minimum lambda value. **(D)** Multivariate Cox regression analysis of 9 prognostic genes from LASSO regression analysis. **(E)** Heatmap of the prognostic signature genes expression profiles for TCGA_LUAD.

**Figure 2:**
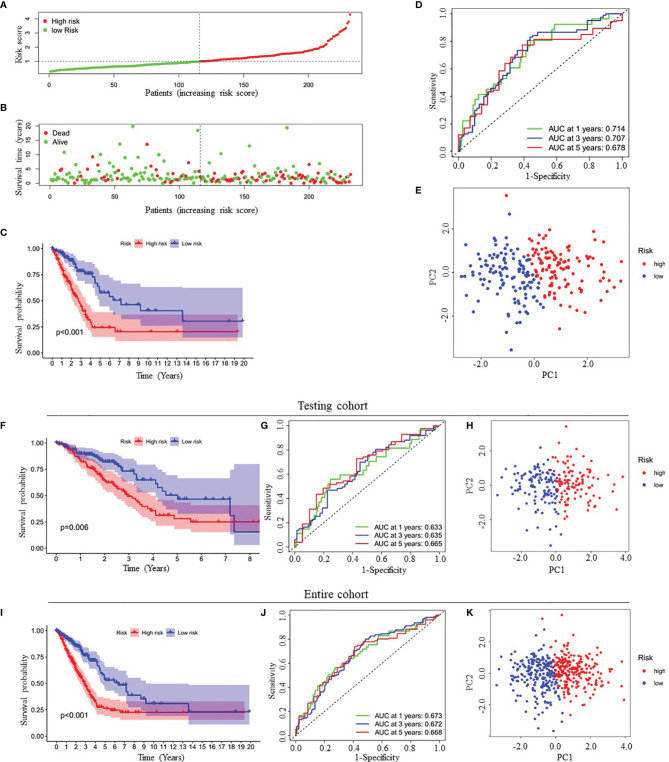
Prognostic analysis of four-gene signature in the training cohort, testing cohort and entire cohort. **(A, B)** The distribution of risk score **(A)** and patient’s survival time **(B)** in the training cohort. **(C, F, I)** Kaplan-Meier survival analysis of the four-gene signature in the training cohort **(C)**, testing cohort **(F)** and entire cohort **(I)**, respectively. **(D, G, J)** Time-dependent ROC analysis of the four-gene signature in the training cohort **(D)**, testing cohort **(G)** and entire cohort **(J)**, respectively. **(E, H, K)** Principal components analysis (PCA) of whole gene expression data between low- and high-risk groups in the training cohort **(E)**, testing cohort **(H)** and entire cohort **(K)**, respectively. ROC, receive operating characteristic; AUC, area under curve.

Because this ceRNA network constructed above is composed of many genes and their interactions, it isn’t easy to clarify its diagnostic and prognostic significance. Therefore, a univariate Cox regression analysis (Hazard Ratio ≠1, p<0.05) was performed to screen the node genes related to the overall survival (OS) of LUAD patients in the ceRNA network using the TCGA_LUAD database. The results showed that 24 node genes were significantly associated with the OS (**Figure 1A** and **Supplementary Table 4**). Subsequently, to reduce the complexity of the risk model, Lasso regression analysis was used to remove genes with relatively lower correlation, and 9 of the 24 prognostic genes were screened out (**Figures 1B, C**). Then, we randomly divided 462 TCGA_LUAD patients with survival data into training and testing cohorts using the R package “caret”. A prognostic signature model was developed in the training cohort based on multivariate Cox regression analysis (**Supplementary Table 5**). Then, four candidate signature genes were identified, namely, GPI, IL22RA1, CCT6A, and SPOCK1. **Figures 1D** **, E** presented the forest plots and heatmap of the four prognostic genes. Furthermore, a 4-gene signature-based risk score formula was constructed as Risk score=0.314∗GPI+0.127∗ IL22RA1+0.330∗CCT6A+0.104∗SPOCK1. According to the median risk scores, 232 LUAD patients in the training cohort were divided into high-risk and low-risk groups. As depicted in **Figures 2A, B**, the increase of risk score was related to the poor OS of patients with LUAD. Kaplan-Meier curve showed that OS decreased in patients in the high-risk group (**Figure 2C**). Time-dependent ROC analysis observed that compared with each of the above genes, the four-gene prognostic signature had larger AUC values (**Figure 2D** and **Supplementary Figure 3**). Moreover, principal component analysis (PCA) clearly identified a significantly different distribution between the two risk groups (**Figure 2E**).

Finally, to verify the predictive value of the four-gene signature, we used the testing cohort (n=230) and the entire cohort (n=462) as the validation set to evaluate the findings from the training cohort. Similar to the results of the training cohort, the KM curves of the two validation sets demonstrated that patients in the low-risk group exhibited better OS (**Figures 2F, I**). The AUC of the four-gene signature was 0.633, 0.635, 0.665, 0.673, 0.672 and 0.668 at the 1-year, 3-year, and 5-year timepoints in the testing cohort and the entire cohort, respectively (**Figures 2G, J**). In addition, PCA also showed similar results as the training cohort (**Figures 2H, K**).

### Verification of the Expression of Prognostic Signature Genes Between LUAD and Adjacent Nonumorous Lung Tissues

**Figure 3:**
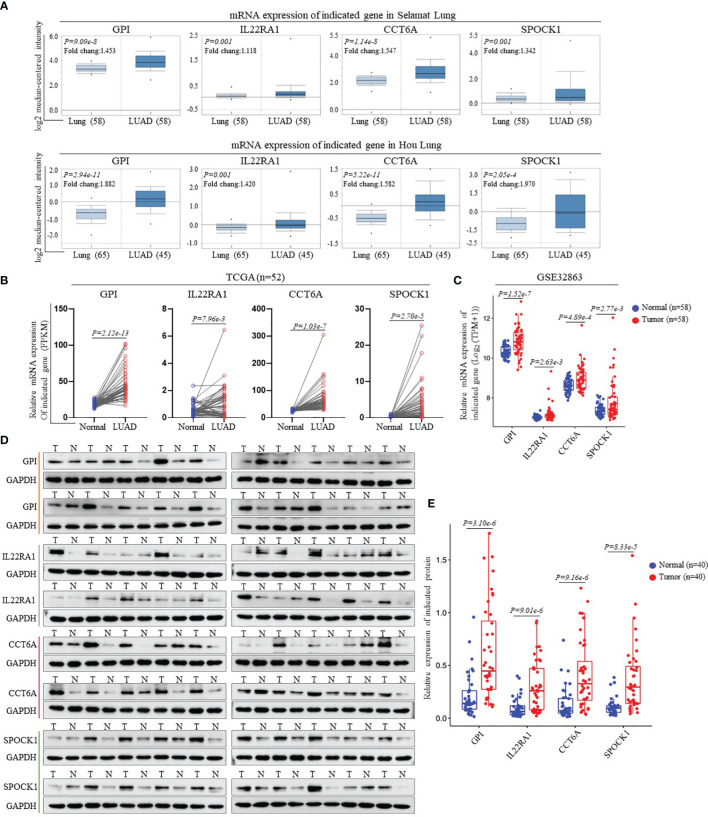
Validation of the gene expression contained in prognostic signature. **(A)** Expression levels of GPI, IL22RA1, CCT6A and SPOCK1 in each study based on the Oncomine database. **(B)** The mRNA expression levels of GPI, IL22RA1, CCT6A and SPOCK1were evaluated using 52 matched LUAD samples from the TCGA_LUAD database. **(C)** The mRNA expression levels of GPI, IL22RA1, CCT6A and SPOCK1were estimated using GSE32863 dataset. **(D, E)** Representative Western blotting analysis of the protein expression levels of GPI, IL22RA1, CCT6A and SPOCK1 in 40 paired LUAD tissues and adjacent nontumor tissues **(D)**. Taking GAPDH as the loading control, the quantitative results of grayscale scanning were displayed **(E)**.

We performed external validation of four prognostic signature genes. The Oncomine database analysis observed that compared to normal lung tissues, the expression levels of GPI, IL22RA1, CCT6A, and SPOCK1 mRNA in LUAD tissues were significantly higher (**Figure 3A**). Moreover, we further confirmed mRNA expression of these four genes in LUAD from TCGA’ paired samples and GSE32863 databases (**Figures 3B, C**). Additionally, we investigated the protein expression levels of these four genes in 40 pairs of LUAD tissues and adjacent nontumorous tissues by Western blot analysis. The results showed that GPI, IL22RA1, CCT6A, and SPOCK1 expression in the tumor tissues (T) was markedly higher than in the control tissues (N) (n=40, *P<0.05*; **Figures 3D, E** and **Supplementary Figure 4**).

### Performance Comparison of the ceRNA Network-Related Genes Signature With Other Reported Gene Signatures in Prognosis Evaluation

**Figure 4:**
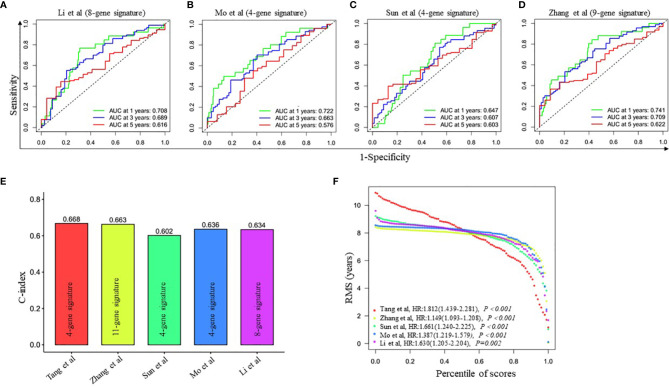
Comparison of the four-gene prognostic signature risk model with other reported risk models. **(A–D)** Time-dependent ROC analysis of four other published gene signatures. **(E)** Concordance index (C-index) of five prognostic risk models. Our prognostic risk model (red histogram) has the highest C-index. **(F)** Restricted mean survival (RMS) time curve of all five risk models.

To further evaluate the prediction performance of the ceRNA network-related genes signature, we selected four other published gene signatures obtained from Li’s (21), Mo’s (22), Sun’s (23) and Zhang’s (24) for comparison. The risk score of each patient was calculated according to the corresponding genes in these four models using the same method (multivariable Cox regression analysis) in the training cohort and then evaluated the time-dependent ROC. **Figure 2D** and **Figures 4A** **–D** revealed that the AUC of the ceRNA network-related gene signature for 5-year OS was 0.678, which was significantly larger than that of Li’s (0.616), Mo’s (0.576), Sun’s (0.603) and Zhang’s (0.622) gene signatures. The C-index of all prognostic signatures calculated by the restricted mean survival (RMS) package showed that our model had the highest C-index with 0.668 (**Figure 4E**). Moreover, the RMS time curve of all five prognostic models also demonstrated that our 4-gene signature performed best at a time period greater than 8 years (**Figure 4F**). These results suggested that the ceRNA network-related genes signature might provide better prognosis evaluation performance for LUAD.

### Construction and Validation of a Predictive Nomogram Based on the Risk Signature

**Figure 5:**
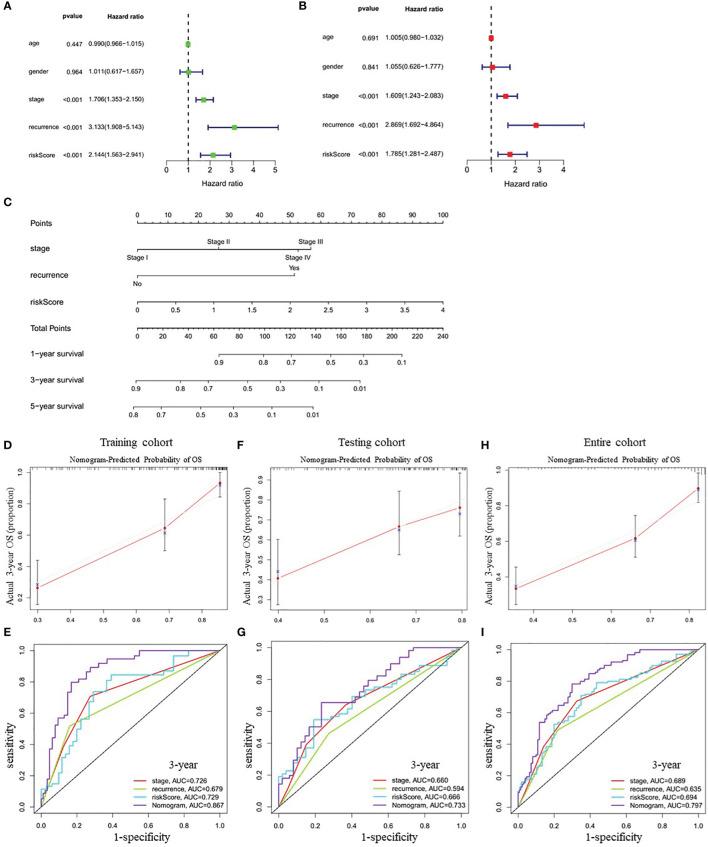
Correlation between indicated variables and prognosis of LUAD patients. **(A, B)** Analyses of correlations between the indicated variables and overall survival of LUAD patients by univariate and multivariate Cox regression, respectively. **(C)** Nomogram for predicting the 1-, 3-, and 5-year overall survival of LUAD patients. **(D, F, H)** The calibration plots for predicting 3-year survival in the training cohort **(D)**, testing cohort **(F)** and entire cohort **(H)**, respectively. **(E, G, I)** The time-dependent ROC curves of the stage, recurrence, risk score and nomogram in 3-year OS prediction in the training cohort **(E)**, testing cohort **(G)** and entire cohort **(I)**, respectively. OS, overall survival; ROC, receiver operating characteristic.

Univariate Cox regression analysis showed that tumor stage (HR=1.706, 95% CI=1.353-2.150, *P<0.001*), recurrence (HR=3.133, 95% CI=1.908-5.143, *P<0.001*), and the risk score (HR=2.144, 95% CI=1.563-2.941, *P<0.001*) were closely correlated with OS in training cohort (**Figure 5A**). Multivariate Cox regression analysis further confirmed the above results (**Figure 5B**). Therefore, these three factors were combined to construct a compound nomogram for predicting the OS of patients with LUAD at 1-, 3- and 5-year (**Figure 5C**). The calibration plot of the nomogram for predicting 3-year OS of LUAD patients in the training cohort showed great consistency between actual observation and nomogram prediction (**Figure 5D**), and the nomogram model’s C-index for prediction of OS was 0.778 (95% CI=0.728-0.829, *P=1.97e-27*). Moreover, the AUC value of the nomogram for predicting 3-year OS was larger than that of the stage, recurrence and risk score, suggesting that using this nomogram to predict OS might bring more net benefit (**Figure 5E**). Finally, we further assessed the predictive value of the four-gene prognostic nomogram using the testing cohort and the entire cohort. The calibration plots (**Figures 5F, H**) and the time-dependent ROC curves of risk score (**Figures 5G, I**) were consistent with the results derived from the training set.

### Functional Annotation of the Risk Signature

**Figure 6:**
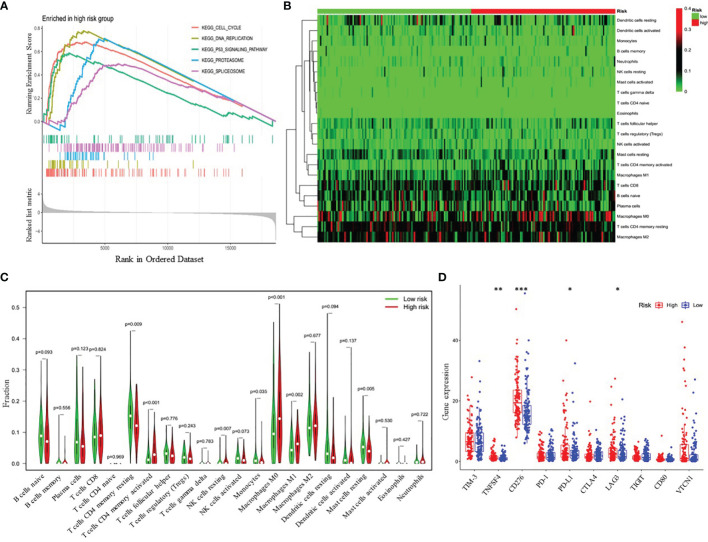
Functional analyses of the high- and low-risk groups. **(A)** Differences in biological functions by GSEA analysis. **(B)** Heatmap of the 22 immune cell types in high- and low-risk groups. **(C)** The fractions of 22 immune cell types estimated with CIBERSORT and the differences between low- and high-risk LUAD patients. **(D)** Differential expression analysis of immune checkpoint genes. *p < 0.05; **p < 0.01; ***p < 0.001.

To further explore the potential biological pathways and processes related to the four-gene signature, we conducted gene set enrichment on samples within the training cohort by GSEA. We found that critical pathways associated with tumorigenesis, including cell cycle, DNA replication, P53 signaling pathway, proteasome, and spliceosome, were significantly enriched in the high-risk group (**Figure 6A** and **Supplementary Table 6**). Additionally, we investigated whether the risk model was related to the tumor immune microenvironment. We plotted the heatmap of 22 tumor-immune cell types, showing the distribution of these immune cells (**Figure 6B**). Then, Wilcoxon rank-sum test was used and revealed that high-risk LUAD patients had significantly higher proportions of T cells CD4 memory activated, NK cells resting, macrophages M0 and macrophages M1 and lower proportions of T cells CD4 memory resting, monocytes, and Mast cells resting (**Figure 6C**). Furthermore, we further analyzed the correlation between the risk groups and the expression of immune checkpoint molecules, including programmed death-1 (PD-1), programmed death-ligand 1 (PD-L1), cytotoxic T-lymphocyte-associated protein 4 (CTLA-4), lymphocyte activation gene-3 (LAG3), T-cell immunoglobulin and mucin-domain containing-3 (TIM-3), T cell immunoreceptor with Ig and ITIM domains (TIGIT), CD80, CD276, tumor necrosis factor superfamily member 4 (TNFSF4), and V-set domain-containing T-cell activation inhibitor-1 (VTCN1). The results showed that compared with the low-risk group, the high-risk group had markedly higher expression levels of TNFSF4, CD274, PD-L1, and LAG3 (**Figure 6D**). Therefore, the heterogeneity of immune cell infiltrations and immune checkpoint molecules expression observed in these results may provide potential prognostic indicators and targets for immunotherapy in patients with LUAD.

### Analysis of Chemotherapeutic Responses Between High- and Low-Risk Patients With LUAD

**Figure 7:**
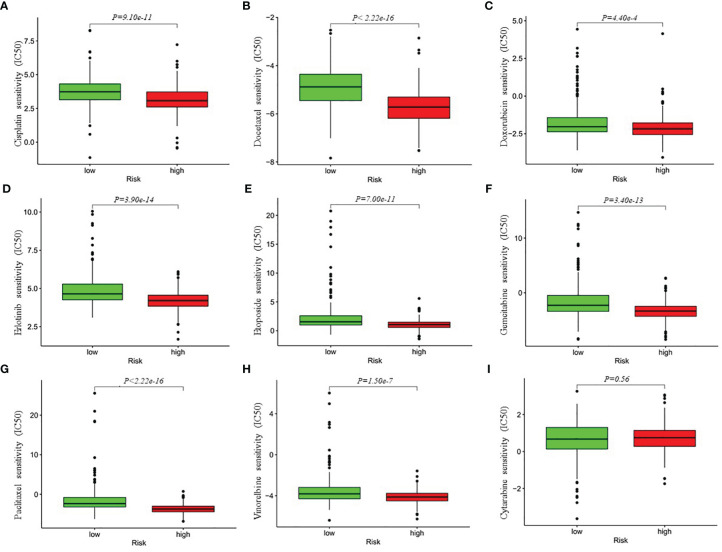
Differential chemotherapeutic responses in low- and high-risk LUAD patients **(A–I)**. The IC50 values of the high- and low-risk groups were calculated based on the GDSC (Genomics of Drug Sensitivity in Cancer) data (https://www.cancerrxgene.org/). IC50, half maximal inhibitory concentration.

Besides immune checkpoint blockades therapy, chemotherapy is still an effective treatment for advanced LUAD patients. Thus, we attempted to investigate the response to the common chemotherapeutic drugs in low- and high-risk patients with LUAD in the entire cohort. The IC50 values of the high- and low-risk groups were calculated based on the GDSC data. The results demonstrated that high-risk LUAD patients showed increased sensitivity to cisplatin, docetaxel, doxorubicin, erlotinib, etoposide, gemcitabine, paclitaxel and Vinorelbine, while there was no significant difference in IC50 value of cytarabine between the high-risk group and low-risk group, which indicated that the four-gene risk model might act as a potential predictor for chemosensitivity (**Figure 7**).

### GPI, IL22RA1, CCT6A and SPOCK1, Which Constitute the Risk Signature, Affect LUAD Prognosis in Association With Activation of PI3K/AKT Signaling Pathway

**Figure 8:**
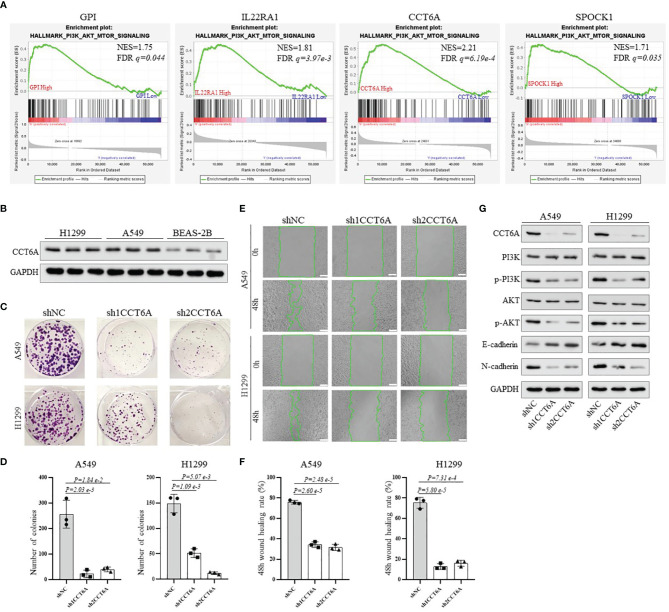
To explore the potential mechanism of the risk model-related genes GPI, IL22RA1, CCT6A and SPOCK1 on the progression of LUAD. **(A)** GSEA analysis was conducted using TCGA_LUAD database to compare the high expression and low expression of GPI, IL22RA1, CCT6A and SPOCK1, respectively. **(B)** Western blotting analysis of the protein expression level of CCT6A in BEAS-2B, A549 and H1299 cells. **(C, D)** The effect of CCT6A silencing on colony formation in A549 and H1299. **(E, F)** The effect of CCT6A knockdown on cell migration was analyzed by wound healing assay in A549 and H1299 (scale bar, 100μm). **(G)** Western blot analysis of indicated protein expression level. GSEA, Gene Set Enrichment Analysis.

Finally, to obtain more mechanistic insights into the impact of GPI, IL22RA1, CCT6A and SPOCK1 on the progression of LUAD, Gene Set Enrichment Analysis (GESA) was performed using TACG_LUAD database to compare the high expression and low expression of GPI, IL22RA1, CCT6A and SPOCK1, respectively. We observed that many important regulatory genes involved in the PI3K-AKT-mTOR signaling pathway, which plays an important role in regulating various oncogenic processes (25), were markedly enriched in cells with high GPI, IL22RA1, CCT6A and SPOCK1 expression (**Figure 8A**). In view of the highest normalized enrichment score (NES) of CCT6A (NES) (NES=2.21, *P= 6.19e-4*), we mainly chose CCT6A to explore the relevant mechanism. We first examined the expression level of CCT6A in BEAS-2B, A549 and H1299 cell lines by Western blot (**Figure 8B**). Due to the high expression level of CCT6A in two non-small cell lung cancer (NSCLC) cell lines, A549 and H1299, we used short hairpin RNA (shRNA) targeting CCT6A to silence CCT6A. The results showed that CCT6A knockdown substantially reduced proliferation and migration of A549 and H1299 (**Figures 8C–F**). Moreover, Western blot analysis revealed that CCT6A silencing significantly inhibited epithelial-mesenchymal transition (EMT) of A549 and H1299, evidenced by a marked reduction in the protein levels of N-cadherin and marked increase of E-cadherin. Notably, we also observed that CCT6A knockdown markedly decreased the protein levels of p-PI3K and p-AKT (**Figure 8G**), suggesting that CCT6A may affect the EMT of LUAD cells by activating PI3K/AKT pathway and then affect the malignant of LUAD and prognosis of LUAD patients. In the future, we will continue to focus on the specific mechanism of GPI, IL22RA1 and SPOCK1 in LUAD.

## Discussion

The ceRNA networks consist of protein-coding mRNAs and non-coding RNAs (ncRNAs), such as miRNAs and lncRNAs (19). After Salmena et al. first proposed the ceRNA hypothesis, increasing investigations regarding ceRNAs have documented that dysregulated ceRNAs play critical roles in cancer initiation and progression. Nevertheless, an extensive analysis of the prognostic value and clinical significance of node genes based on lncRNA-mediated ceRNA network in LUAD samples has not been reported. The present study is the first systematic analysis of the expression levels of 117 node genes of the ceRNA network in LUAD. Then, according to the multi-step selection, a prognostic risk model significantly associated with the survival rate, chemotherapeutic responses and tumor immune microenvironment of LUAD patients was constructed based on four the ceRNA network-related candidate signature genes.

Given the functional interdependence between the different RNA molecules, in recent years, the focus of cancer pathology research has shifted from individual RNAs carrying cancer-related dysregulation to network-based potential mechanisms and clinical significance, such as the lncRNA-mediated ceRNA network hypothesis. In 2018, Fang et al. reported a lncRNA-miRNA-mRNA network, which was demonstrated to be strongly associated with certain clinical characteristics of human head and neck squamous cell carcinoma (26); In addition, Liu et al. comprehensively investigated the gain and loss of ceRNAs in prostate cancer (PC) and proposed its role in identifying potential biomarkers and treatment options for PC (27). In this study, we used bioinformatics analysis to identify the ceRNA networks, which could regulate the survival and prognosis of LUAD patients through 95 protein-coding mRNAs, 7 lncRNAs, and 15 miRNAs. In the ceRNA networks, 24 node genes, such as CPS1, DEPDC1, MYBL2, MYBL2, LOXL2, etc., were significantly associated with overall survival in LUAD. According to the hypergeometric testing and correlation analysis, the results of the ceRNAs network revealed that GPI, IL22RA1, CCT6A, and SPOCK1 were prognostic signature genes used to construct the prognostic risk model. Subsequently, the correlation analysis between the risk model and the survival rate, immune cell infiltration, the expression of immune checkpoint genes and chemotherapeutic responses of LUAD patients discovered that this four-gene signature could provide a new method for evaluating LUAD patients, guiding prognosis prediction and the choice of immunotherapy and chemotherapy.

Prior work has reported these model genes’ biological function and expression patterns. GPI (glucose-6-phosphate isomerase) is a housekeeping cytoplasmic enzyme. The expression of GPI is induced by HIF-1 (28, 29) and c-Myc (30) and is frequently up-regulated in many types of cancer (31). GPI catalyzes the interconversion between glucose-6-phosphate and fructose-6-phosphate and plays a vital role in glycolytic and gluconeogenic pathways. Besides its role as a glycolytic enzyme, mammalian GPI can function as a tumor-secreted cytokine and an angiogenic factor that stimulates endothelial cell motility (32). IL22RA1 (interleukin-22 receptor subunit α-1) is a component of IL20, IL22 and IL24 receptors. The IL22 receptor formed by IL22RA1 and IL10RB realizes IL22 signal to activate Signal Transducers and Activators of Transcription (STATs), nuclear factor kappa B (NF-κB), mitogen-activated protein kinase (MAPK) and phosphatidylinositide 3-kinase-Akt mammalian target of rapamycin (PI3K-Akt-mTOR) pathways (33, 34), modulates a variety of biological properties closely related to tumorigenesis, development and metastasis, such as inflammation, mitosis, proliferation, survival, apoptosis and angiogenesis (35). CCT6A (chaperonin containing TCP1 subunit 6A) is one of eight subunits of the critical molecular chaperone, T-complex protein 1 Ring complex (TRiC). It is estimated that TRiC can directly help fold up to 10% of cytosolic proteins (36, 37) and provide the unique ability to fold certain proteins that simpler chaperone systems cannot fold. This strict requirement of TRiC is essential for folding critical proteins involved in tumorigeneses, such as tumor suppressor Von Hippel-Lindau (VHL) (38), p53 (39) and the pro-oncogenic protein STAT3 (40). Recently, Hallal et al. observed that the expression level of CCT6A was markedly increased in glioblastoma patients, and its expression was associated with EGFR, speculating that CCT6A might be a potential biomarker of glioblastoma with prognostic significance (41). SPOCK1 (SPARC/osteonectin, cwcv and kazal-like domains proteoglycan 1), also referred to as testincan-1, is a crucial regulator of the dynamic balance of extracellular matrix (ECM) and mediates epithelial-to-mesenchymal transition (EMT) in cancer cells. It can activate many molecular signaling pathways, such as Wnt/β-catenin (42), EMT process (43), and mTOR/S6K (44) signaling pathways, leading to ECM remodeling, cancer cell proliferation and invasion, but inhibiting cell apoptosis. Moreover, Miao et al. found that lung cancer patients with high SPOCK1 expression have decreased life expectancy compared to those with low expression (42). Importantly, we verified that GPI, IL22RA1, CCT6A, and SPOCK1 were overexpressed in 40 pairs of LUAD tissues and adjacent normal lung tissues by Western blotting analysis.

The PI3K/AKT signaling pathway, which plays a crucial role in regulating cell growth, differentiation, apoptosis and metastasis, is frequently activated in multiple human cancer, including lung adenocarcinoma (45, 46). Moreover, recent studies have demonstrated that activation of the PI3K/AKT signaling pathway can induce EMT, which is usually considered to be an activator of cancer progression (47, 48). In this study, we found that the high expression of GPI, CCT6A, IL22RA1 and SPOCK1 was closely related to the activation of PI3K/AKT pathway through GSEA analysis, while CCT6A knockdown caused reduced phosphorylation of PI3K/AKT, increased expression of E-cadherin and decreased N-cadherin. We speculated that PI3K/AKT signaling pathway activation might be a critical process involved in the progression of GPI, CCT6A, IL22RA1 and SPOCK1-overexpression tumors. Our findings indicate that these genes may play critical roles in the progression of LUAD. Therefore, it is necessary to further study the biological functions of these four genes in LUAD. Our current research is mainly based on public databases and limited clinical tissue specimens of LUAD. In the future, additional studies employing retrospective design are required to verify the robustness and reproducibility of this four-gene signature.

In conclusion, An LUAD-specific lncRNA-mediated ceRNA network was constructed by bioinformatics methods. Then, a four-gene prognostic signature was developed based on node genes of this network, demonstrating high performance in predicting the survival and chemotherapeutic responses of LUAD patients. Finally, independent prognostic factors were further analyzed and combined into a well-executed nomogram that showed strong potential for clinical applications.

## Data Availability Statement

The original contributions presented in the study are included in the article/**Supplementary Material**. Further inquiries can be directed to the corresponding author.

## Ethics Statement

The studies involving human participants were reviewed and approved by the Ethics Committee of Medical School of Henan University, China. Informed consent was obtained from each patient included in the study.

## Author Contributions

FL conceived and designed this article. WT, QL, JZ, and XZ participated in the experimental data collection. NF provided technical assistance. FL drafted the manuscript. SJ revised the study draft. All authors contributed to the article and approved the submitted version.

## Funding

This work was supported by the National Natural Science Foundation of China (No: 81372147) and Henan University support grant CX3070A0780502.

## Conflict of Interest

The authors declare that the research was conducted in the absence of any commercial or financial relationships that could be construed as a potential conflict of interest.

## Publisher’s Note

All claims expressed in this article are solely those of the authors and do not necessarily represent those of their affiliated organizations, or those of the publisher, the editors and the reviewers. Any product that may be evaluated in this article, or claim that may be made by its manufacturer, is not guaranteed or endorsed by the publisher.
