# Supplementary material for: Identification of a Prognostic Signature Composed of GPI, IL22RA1, CCT6A and SPOCK1 for Lung Adenocarcinoma Based on Bioinformatic Analysis of lncRNA-Mediated ceRNA Network and Sample Validation
Source: Front Oncol. 2022 Mar 28;12:844691. doi: 10.3389/fonc.2022.844691 (PMC9012227; doi:10.3389/fonc.2022.844691)
Supplement: Supplementary file 1 [file DataSheet_1.pdf]

Supplementary Material

1 Supplementary Figures and Table

1.1 Supplementary Figures

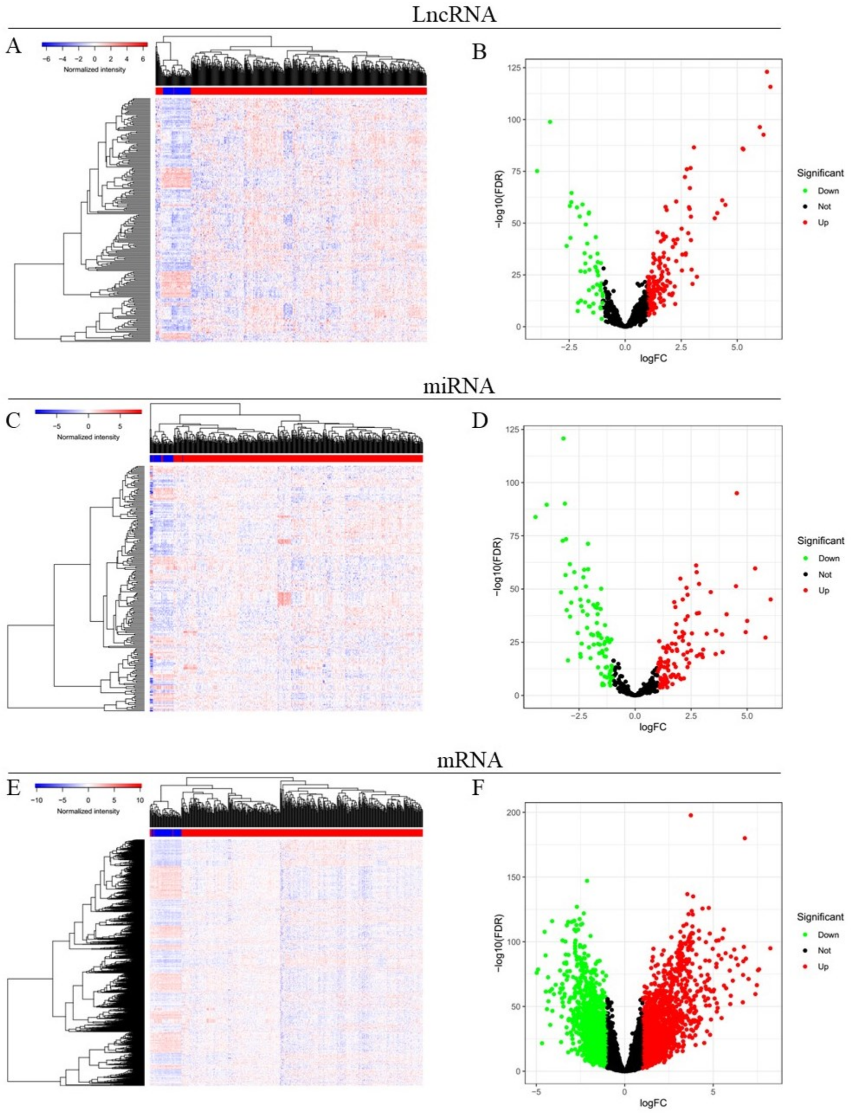

Supplementary Figure 1. Identification of differentially expressed lncRNA, miRNA and mRNA in 497 lung adenocarcinoma (LUAD) and 54 adjacent nontumor tissues. (A, C, E) The heatmaps of genome-wide differentially expressed lncRNA (A), miRNA (C) and mRNA (E) in the TCGA_LUAD database. (B, D, F) Volcano plots of differentially expressed lncRNA (B), miRNA (D) and mRNA (F)

in TCGA_LUAD database. Red and green represent upregulated and downregulated genes, respectively.

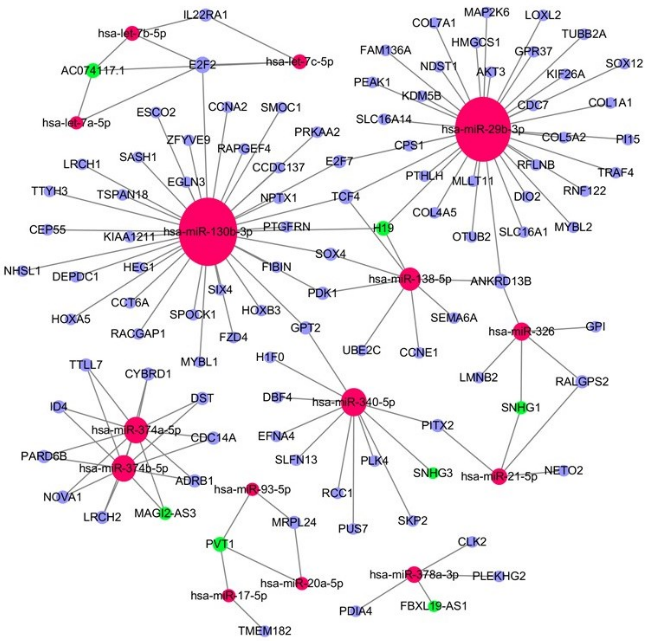

Supplementary Figure 2. LncRNA-miRNA-mRNA network constructed from differentially expressed lncRNA, miRNA and mRNA in TCGA_LUAD database. Red, green and blue represent miRNA, lncRNA and mRNA, respectively.

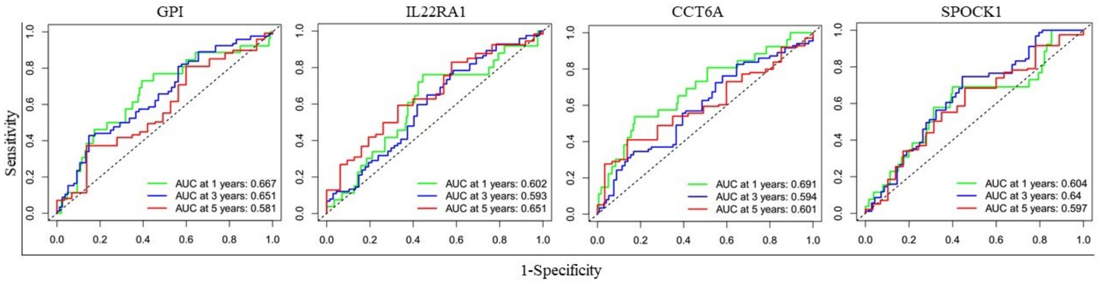

Supplementary Figure 3. Time-dependent receiver operating characteristic (ROC) analysis of the sensitivity and specificity of overall survival (OS) for GPI, IL22RA1, CCT6A and SPOCK1 in the training cohort. AUC, area under curve.

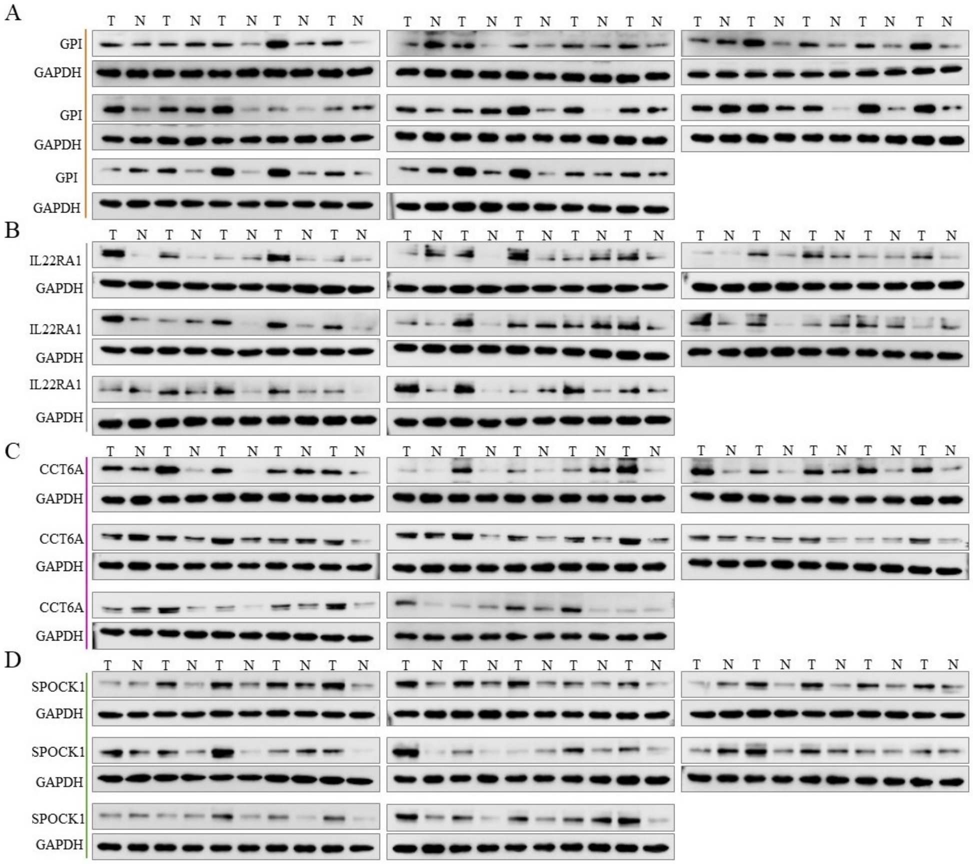

Supplementary Figure 4. Expression levels of GPI (A), IL22RA1 (B), CCT6A (C) and SPOCK1 (D) proteins in 40 paired LUAD and adjacent nontumor tissues were analyzed by Western blot.

1.2 Supplementary Table

Supplementary Table 1. The antibodies used in this study

Antibodies	Catalog#	Source
anti-GPI (AMF)	#57893	Cell Signaling Technology
anti-GAPDH	60004-1-Ig	Proteintech
anti- IL22RA1	ab5984	Abcam
anti- CCT6A	ab110905	Abcam
anti- SPOCK1	ab229935	Abcam
anti-PI3K	#4249	Cell Signaling Technology
anti-p-PI3K(Y458)	ab278545	Abcam
anti-AKT	60203-2-Ig	Proteintech
anti-p-AKT(Ser473)	#4060	Cell Signaling Technology
anti-E-cadherin	#14472	Cell Signaling Technology
anti-N-cadherin	#14215	Cell Signaling Technology
