# Supplementary material for: Identification of a Prognostic Signature Composed of GPI, IL22RA1, CCT6A and SPOCK1 for Lung Adenocarcinoma Based on Bioinformatic Analysis of lncRNA-Mediated ceRNA Network and Sample Validation
Source: Front Oncol. 2022 Mar 28;12:844691. doi: 10.3389/fonc.2022.844691 (PMC9012227; doi:10.3389/fonc.2022.844691)
Supplement: Supplementary file 2 [file Table_1.docx]

**Supplementary Table 1**. The antibodies used in this study

| **Antibodies** | **Catalog#** | **Source** |
| --- | --- | --- |
| anti-GPI (AMF) | #57893 | Cell Signaling Technology |
| anti-GAPDH | 60004-1-Ig | Proteintech |
| anti- IL22RA1 | ab5984 | Abcam |
| anti- CCT6A | ab110905 | Abcam |
| anti- SPOCK1 | ab229935 | Abcam |
| anti-PI3K | #4249 | Cell Signaling Technology |
| anti-p-PI3K(Y458) | ab278545 | Abcam |
| anti-AKT | ab8805 | Abcam |
| anti-p-AKT(Ser473) | #4060 | Cell Signaling Technology |
| anti-E-cadherin | #14472 | Cell Signaling Technology |
| anti-N-cadherin | #14215 | Cell Signaling Technology |
